# A Conserved Role for Asrij/OCIAD1 in Progenitor Differentiation and Lineage Specification Through Functional Interaction With the Regulators of Mitochondrial Dynamics

**DOI:** 10.3389/fcell.2021.643444

**Published:** 2021-07-06

**Authors:** Arindam Ray, Kajal Kamat, Maneesha S. Inamdar

**Affiliations:** Molecular Biology and Genetics Unit, Jawaharlal Nehru Centre for Advanced Scientific Research, Bengaluru, India

**Keywords:** mitochondrial dynamics, blood progenitor differentiation, blood lineage choice, progenitor heterogeneity, Asrij, Notch signaling, *Drosophila* lymph gland, human embryonic stem cells (hESC)

## Abstract

Mitochondria are highly dynamic organelles whose activity is an important determinant of blood stem and progenitor cell state. Mitochondrial morphology is maintained by continuous fission and fusion and affects stem cell proliferation, differentiation, and aging. However, the mechanism by which mitochondrial morphology and dynamics regulate cell differentiation and lineage choice remains incompletely understood. Asrij/OCIAD1 is a conserved protein that governs mitochondrial morphology, energy metabolism and human embryonic stem cell (hESC) differentiation. To investigate the *in vivo* relevance of these properties, we compared hESC phenotypes with those of *Drosophila* hematopoiesis, where Asrij is shown to regulate blood progenitor maintenance by conserved mechanisms. In concordance with hESC studies, we found that *Drosophila* Asrij also localizes to mitochondria of larval blood cells and its depletion from progenitors results in elongated mitochondria. Live imaging of *asrij* knockdown hemocytes and of OCIAD1 knockout hESCs showed reduced mitochondrial dynamics. Since key regulators of mitochondrial dynamics actively regulate mitochondrial morphology, we hypothesized that mitochondrial fission and fusion may control progenitor maintenance or differentiation in an Asrij-dependent manner. Knockdown of the fission regulator Drp1 in *Drosophila* lymph gland progenitors specifically suppressed crystal cell differentiation whereas depletion of the fusion regulator Marf (*Drosophila* Mitofusin) increased the same with concomitant upregulation of Notch signaling. These phenotypes were stronger in anterior progenitors and were exacerbated by Asrij depletion. Asrij is known to suppress Notch signaling and crystal cell differentiation. Our analysis reveals that synergistic interactions of Asrij with Drp1 and Marf have distinct impacts on lymph gland progenitor mitochondrial dynamics and crystal cell differentiation. Taken together, using invertebrate and mammalian model systems we demonstrate a conserved role for Asrij/OCIAD1 in linking mitochondrial dynamics and progenitor differentiation. Our study sets the stage for deciphering how regulators of mitochondrial dynamics may contribute to functional heterogeneity and lineage choice in vertebrate blood progenitors.

## Introduction

In addition to their well-established role in energy metabolism, recent studies show that mitochondria act as a critical regulatory hub of signaling and contribute to stem and progenitor survival and cell fate decisions in pluripotent embryonic stem cells (ESCs) or multipotent hematopoietic stem cells (HSCs) (Bejarano-Garcia et al., 2016; Anso et al., 2017; Zhang et al., 2018). Dynamicity of the mitochondrial network governs mitochondrial function and cell fate specification (Liesa and Shirihai, 2013; Ni et al., 2015; Wai and Langer, 2016; Seo et al., 2018). Balanced mitochondrial fission and fusion maintains mitochondrial quality control through segregation of damaged mitochondria or exchange of components, electrochemical gradients, and metabolites (Twig et al., 2008; van der Bliek et al., 2013; Liu et al., 2020). Mitochondrial morphology and dynamics vary across cell states, lineages, and tissues. Stem and progenitor cells contain fragmented mitochondria with immature cristae, while differentiated cells generally have longer mitochondria with mature ultrastructure (Khacho et al., 2016; Seo et al., 2018). Mitochondria in HSCs also undergo fragmentation upon differentiation to lineage committed progenitors (Luchsinger et al., 2016).

Mitochondrial membrane remodeling proteins actively control mitochondrial dynamics to shape the mitochondrial network through regulation of fission, fusion, biogenesis and degradation. Dynamin related protein 1 (Drp1) is a GTPase that acts as the key mediator of fission and segregation of the mitochondrial network whereas Mitofusins (Mfn1/2) are the main membrane bound GTPases that promote mitochondrial outer membrane fusion (Seo et al., 2018). Other proteins such as Opa1, Fis1, Mid49/51, etc., also regulate various other steps of mitochondrial fission and fusion (Atkins et al., 2016; Wai and Langer, 2016; Tilokani et al., 2018). Many signaling pathways including calcium, ROS, and Notch signaling, which are essential for cell fate decisions depend on the fission-fusion machinery. Drp1 can act in a positive feedback loop with Notch signaling in triple negative breast cancer cells (Chen et al., 2018). Inhibition of Mitofusin2 can upregulate Notch signaling through Calcineurin A in mouse embryonic stem cells (Kasahara et al., 2013). Recent reports highlight the importance of balanced Drp1 or Mitofusin activity in determining HSC fate decisions such as lineage-biased differentiation potential (Luchsinger et al., 2016; Hinge et al., 2020). Drp1 maintains HSC regenerative potential by establishing divisional memory and regulates myeloid lineage reconstitution (Hinge et al., 2020). Also, Mfn2 maintains HSCs with extensive lymphoid potential through inhibition of excessive calcium-dependent NFAT (Nuclear Factor of Activated T-cells) signaling, probably by tethering mitochondria to the endoplasmic reticulum (Luchsinger et al., 2016). Drp1 and Mfn2 may impact various developmental processes in different ways, due to their opposite roles in mitochondrial dynamics (Sandoval et al., 2014). Such correlation of mitochondrial shape to cell fate suggests a possible role of mitochondrial network architecture during hematopoiesis. Despite reports suggesting functional links between mitochondrial dynamics regulators and HSC fate, the mechanism by which they regulate lineage-biased signaling and differentiation is not fully elucidated.

Asrij/OCIAD1 (Ovarian Carcinoma Immunoreactive Antigen Domain containing 1), a conserved regulator of differentiation, localizes to mitochondria in human embryonic stem cells (hESCs) and negatively regulates mitochondrial Complex I activity (Shetty et al., 2018). Depletion of OCIAD1 leads to elongation of mitochondria and increased early mesodermal progenitor formation indicating that OCIAD1 possibly regulates mitochondrial dynamics to influence mitochondrial activity and cellular differentiation (Shetty et al., 2018; Praveen et al., 2020). The *Drosophila* ortholog of OCIAD1, Asrij maintains blood progenitors in the larval hematopoietic organ, the lymph gland. Asrij expression is restricted to the hematopoietic system (Inamdar, 2003). Loss of Asrij causes precocious differentiation to crystal cells, a lineage that is specified by Notch activation (Kulkarni et al., 2011; Khadilkar et al., 2014). Proteomic analysis showed reduced Drp1 levels in *asrij* null lymph glands (Sinha et al., 2019b). Although OCIAD1 controls mitochondrial morphology in hESCs, its genetic interaction with the canonical mitochondrial dynamics regulatory machinery remains unexplored, especially *in vivo*. Hence, we used *Drosophila* larval hematopoiesis as an accessible *in vivo* model to explore whether Asrij regulates mitochondrial dynamics for progenitor maintenance and cell fate decisions.

The *Drosophila* larval lymph gland is a linearly arranged multi-lobed hematopoietic organ that allows analysis of different stages of blood development in a single animal (Rodrigues et al., 2021). This makes it an excellent model to study conserved mechanisms of blood cell homeostasis and function (Jung et al., 2005; Banerjee et al., 2019). The lobes are arranged in pairs along the antero-posterior axis. The anterior-most or primary lobes are well-characterized and demarcated into distinct zones with the peripheral differentiated hemocytes (cortical zone), inner blood progenitors (medullary zone) and a hematopoietic niche (posterior signaling center) (Banerjee et al., 2019). We recently showed that secondary, tertiary, and quaternary lobes, collectively called the posterior lobes, constitute the major part of the progenitors and persist till the end of the larval life (Rodrigues et al., 2021). Blood progenitor cells in the lymph gland have diverse origins and distinct functions and are identified by expression of domeless, TepIV, or E-Cadherin. Unlike anterior progenitors, posterior progenitors are refractile to immune challenge. Blood progenitor diversity is an essential element of the immune response and leads to functional compartmentalization such that younger posterior blood progenitors are maintained as a reserve pool (Rodrigues et al., 2021).

Hemocytes in *Drosophila* are analogous to the myeloid lineage and are of three types- macrophage-like plasmatocytes (identified by expression of P1), crystal cells that melanize (identified by ProPO (Prophenoloxidase) expression) and large lamellocytes (identified by Phalloidin staining for F-actin) that encapsulate foreign bodies such as parasitoid wasp eggs (Banerjee et al., 2019; Cho et al., 2020; Tattikota et al., 2020). Lineage specification is achieved by controlled activation of distinct signaling pathways to maintain hematopoietic homeostasis.

In this study, we elucidate the role of Asrij in mitochondrial dynamics and show that Asrij regulates remodeling of the mitochondrial network in concert with canonical mitochondrial dynamics regulators *Drosophila* Drp1 and Mfn (Mitochondria Assembly Regulatory Factor/Marf), to regulate Notch signaling and blood cell homeostasis. Moreover, our analyses of mitochondrial dynamics across all the progenitor subsets reflects heterogeneity and developmental diversity of such sub-populations. We establish a functional link of Asrij to canonical mitochondrial dynamics regulators in lineage-biased hematopoiesis that will help elucidate the conserved role of this interaction in influencing cell fate decisions.

## Materials and Methods

### Fly Stocks

Canton-S was used as wild type fly strain. *w1118* was used as background control for *arj9/arj9* whereas *e33CGal4* (K. Anderson, Memorial Sloan Kettering Center) was used as parental control for *asrij* knockdown and overexpression. For progenitor-specific knockdown, *domeGal4 UAS 2xEGFP/FM7a* or *domeGal4/FM7b* (Utpal Banerjee, UCLA) was used as driver and parental control. Other stocks used are as follows: *UAS arj RNAi* (VDRC 6633), *UAS arj*, *UAS mito-GFP* (BDSC 8442), *UAS Drp1 RNAi* (BDSC 44155), *UAS Marf RNAi* (BDSC 31157), *UAS Drp1* (BDSC 51647), *UAS Marf* (BDSC 67157), *NRE-GFP/CyO* (BDSC 30727), and *UAS mCD8 RFP* (BDSC 27399).

### Immunostaining Analysis

Third instar larvae were dissected in PBS to prepare lymph gland samples as described before (Khadilkar et al., 2014). Samples were fixed with 4% paraformaldehyde (PF) for 20 min at room temperature (25°C), permeabilized with 0.3% PTX (Triton X-100 in PBS) and incubated in 20% goat serum before primary antibody addition. Antibodies used were mouse anti-COX IV (Abcam, United Kingdom), rabbit anti-Asrij (Kulkarni et al., 2011), mouse anti-P1 (Istvan Ando, BRC Hungary), mouse anti-ProPO, rabbit anti-dsRed (Takara, Japan), and chick anti-GFP (Abcam, United Kingdom).

For hemocyte immunostaining, larvae were bled to extract hemolymph into warm Schneider’s serum-free media (Thermo Fisher Scientific, Waltham, MA, United States). Hemocytes were placed on coverslips to allow attachment for 10 min, then fixed with 4% PF and permeabilized with 0.4% NP40, blocked with 20% goat serum and incubated in primary antibody.

Secondary antibodies used were conjugated to Alexa-Fluor 488, 568, or 633 (Life Technologies, Carlsbad, CA, United States). Phalloidin conjugated to Alexa 568 or 633 (Life Technologies, Carlsbad, CA, United States) was used to visualize lamellocytes. Lymph glands were mounted on coverslips in DAPI-glycerol media. Images were acquired using Zeiss LSM510 Meta or LSM880 confocal microscope in either normal confocal mode or airy scan mode.

### MitoTracker Staining

Hemocytes, attached to coverslips, were incubated with Mitotracker Deep Red (Thermo Fisher Scientific, Waltham, MA, United States) diluted to 200 nM in serum-free Schneider’s media for 20 min at room temperature in the dark. Mitotracker was then washed off with serum-free Schneider’s media and hemocytes fixed in 4% PF. Images were acquired in Zeiss LSM510 Meta microscope at 633 nm excitation.

### Live Imaging of Mitochondria

Mito-GFP expressing hemocytes from larval hemolymph were left to attach onto coverslips in serum-free Schneider’s media for 10 min at 25°C (Standard experimental temperature). The hemocytes were imaged on Zeiss LSM880 confocal microscope with temperature maintained at 25°C with 5% CO_2_. Images were captured every 10 s. Auto-focus module was used to adjust focal plane variation during imaging.

### Quantification

#### Mitochondria Quantification

Co-localization was analyzed using Zen software co-localization tool. Various parameters of mitochondrial network such as branch length, number of branches, number of junctions, and mitochondrial footprint in hemocytes and lymph gland progenitors were quantified using MiNA plugin of Fiji software following protocol described in Valente et al. (2017). Imaris software was used to make 3D reconstruction of mitochondrial surface and quantify number of surfaces and average volume per surface as a readout of aggregation in hemocytes.

Dynamics of mitochondrial network was estimated by quantifying variance of different parameters over time as shown in Hinge et al. (2020). Similar analyses of mitochondrial parameters were performed to assess mitochondrial dynamics in wild type (BJNhem20), OCIAD1 depleted (OCIAD1-Het-KO), and overexpressing (OCIAD1-OV) hESCs (Shetty et al., 2018).

#### Quantification of Hemocytes in Lymph Gland

Progenitor and plasmatocyte fraction in each lymph gland lobe was quantified using Imaris. Briefly, the number of spots (DAPI positive nuclei with >2 μm diameter) close to the reconstructed dome >2xEGFP (for prohemocytes) or P1 (for plasmatocytes) surface, by a set threshold distance (1 μm for prohemocytes and 2 μm for plasmatocytes), was quantified and divided by total number of nuclei. Number of crystal cells and number of cells with high NRE-GFP expression in each lobe was quantified manually and its fraction was calculated in each lobe by dividing with the number of nuclei. Lamellocytes were identified based on large or elongated morphology as revealed by Phalloidin staining. All images within a given figure panel were adjusted equally for brightness and contrast using Adobe Photoshop CS5 extended. Graphs for all figure panels were prepared using GraphPad Prism version 8. BioRender was used to draw cells in the schematic in Figure 5.

Each larva was considered as a biological replicate. Data from each lymph gland lobe was individually considered for quantitation in all graphs. One-way ANOVA or Student’s *t*-test was performed for statistical analysis of data. For datasets with unequal variance across groups, non-parametric tests such as Kruskal Wallis test or Mann-Whitney test was performed.

## Results

### Mitochondrial Morphology Reflects Larval Blood Progenitor Heterogeneity in *Drosophila*

**FIGURE 1:**
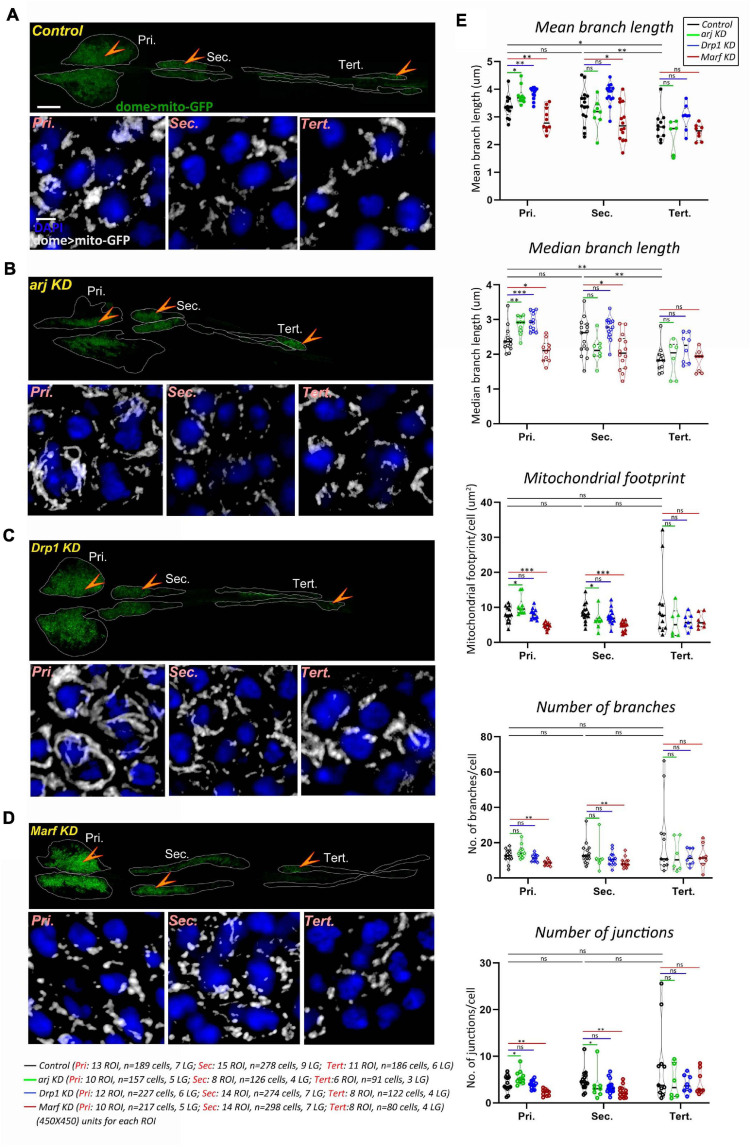
Asrij, Drp1, and Marf regulate mitochondrial morphology in blood progenitors of *Drosophila* lymph gland. **(A–D)** Mitochondria in lymph gland progenitors (pro-hemocytes) of the primary, secondary, and tertiary lobes are marked by *dome* > *mito-GFP* in control (*domeGal4*/ + *; UAS mito-GFP*/ + *;* + / +) **(A)**, *arj KD* (*domeGal4*/ + *; UAS mito-GFP*/ + *;UAS arj RNAi*/ +) **(B)**, *Drp1* KD (*domeGal4*/ + *; UAS mito-GFP*/ + *;UAS Drp1 RNAi*/ +) **(C)**, and *Marf* KD (*domeGal4*/ + *; UAS mito-GFP*/ + *;UAS Marf RNAi*/ +) **(D)** lymph glands. Arrowheads indicate the dome > mito-GFP positive progenitors across different lobes that are shown magnified in the lower panel (Pri.: Primary, Sec.: Secondary, and Tert.: Tertiary). Images represent single confocal section of 0.5 μm for easy visualization of mitochondria. **(E)** Violin plots show quantification of mitochondrial mean and median branch length, footprint, number of branches and number of junctions across primary, secondary, and tertiary lobes. Scale bar: 100 μm for upper LG panel and 5 μm for lower magnified view panel. Kruskal Wallis test was performed to determine statistical significance. ^∗^*P* < 0.05, ^∗∗^*P* < 0.01, ^∗∗∗^*P* < 0.001, ns: non-significant.

Mitochondria are reported to affect progenitor maintenance in the larval lymph gland primary or anterior lobe but mitochondrial morphology and dynamics have not been investigated in blood progenitors. We recently showed that the larval blood progenitor pool is heterogeneous and arranged linearly, with younger progenitors in the posterior lobes (Rodrigues et al., 2021). A comprehensive analysis of mitochondrial morphology (see methods) in the dome + lymph gland progenitors of primary, secondary, and tertiary lobes, using the *domeGal4* driver and the mitoGFP reporter (domeGal4/+; UAS mito-GFP/+; +/+) showed that while primary and secondary lobe progenitors have similar mitochondrial morphology, tertiary lobes have relatively shorter mitochondria. Other parameters such as mitochondrial footprint, number of branches and junctions remained unchanged across progenitor subsets (Figures 1A,E). This is in agreement with the anterior-posterior developmental and functional heterogeneity of progenitors reported earlier (Rodrigues et al., 2021) and indicates that younger progenitors have less mature mitochondria.

### Asrij Regulates Mitochondrial Morphology in *Drosophila* Blood Progenitors and Hemocytes

Several reports show mitochondrial localization of OCIAD1, the human ortholog of Asrij and its interaction with various components of the electron transport chain (ETC) and mitochondrial dynamics machinery (Floyd et al., 2016; Lee et al., 2017; Shetty et al., 2018; Antonicka et al., 2020). OCIAD1 regulates ETC Complex I activity in mitochondria as well as the mitochondrial network architecture (Shetty et al., 2018). Owing to a conserved role in stem cell maintenance and hematopoiesis, we hypothesized that Asrij may similarly regulate mitochondrial features in *Drosophila.*

Immunolocalization analysis for Asrij in *domeGal4*/ + *; UAS mito-GFP*/ + lymph glands showed mitochondrial localization of Asrij in progenitors (Supplementary Figure 1A). Further using the mitochondrial marker COXIV as well as by staining with Mitotracker in Canton(S) hemocytes, we showed that Asrij also localizes to mitochondria in circulating hemocytes (Supplementary Figure 1B).

Depletion of OCIAD1 in hESCs was shown to increase mitochondrial branch length, footprint, and branch number, indicating a shift of dynamics toward enhanced mitochondrial biogenesis and fusion (Shetty et al., 2018). *Asrij* knockdown (*domeGal4*/+; *UAS mito-GFP*/+; *UAS arj RNAi*/ +) in lymph gland progenitors resulted in elongated mitochondria (interpreted through increase in mean and median branch length) (Figures 1A,B,E). In addition, mitochondrial footprint, and number of mitochondrial junctions per cell were increased in primary lobe progenitors, indicating a shift of mitochondrial dynamics toward reduced fission or enhanced fusion. Hence, we conclude that Asrij regulates mitochondrial dynamics in anterior progenitors. However, there was a mild effect on secondary lobes (reduced mitochondrial footprint and junctions) and no significant effect on tertiary lobes. This indicates heterogeneity in dome + progenitor response from anterior to posterior and also suggests that mitochondria in younger progenitors are less sensitive to perturbations (Figures 1A,B,E).

We also examined the mitochondrial network in *Drosophila* circulating hemocytes as these are single cells amenable to high-resolution imaging. Immunostaining for COXIV showed that *asrij* null mutant (*arj9/arj9)* hemocytes had higher mitochondrial branch length, footprint (content), number of branches, and number of junctions as compared to control (*w1118*) (Supplementary Figure 1C). This indicates elongation of mitochondria, poor fission or hyperfusion and increase in mitochondrial content upon loss of Asrij in hemocytes. Additionally, we observed increase in mitochondrial aggregation (as interpreted from increase in mean volume per surface) without significant decrease in the number of mitochondrial clusters in *arj9/arj9* hemocytes (Supplementary Figure 1D).

OCIAD1 overexpression leads to reduction of the mitochondrial footprint and branch length in hESCs (Shetty et al., 2018). While most mitochondrial network parameters (mean and median branch length, number of junctions and mitochondrial aggregation) remained unchanged upon overexpression of Asrij in hemocytes (*e33cGal4/UAS asrij*) there was a significant reduction in number of mitochondria (branches and surfaces) and the mitochondrial footprint (Supplementary Figures 2A,B). This suggests that regulatory mechanisms operating to control mitochondrial dynamics in *Drosophila* hemocytes are Asrij-dependent. Taken together, our data show functional conservation of Asrij in controlling mitochondrial morphology and network architecture.

### Anterior Progenitors Are More Sensitive to Perturbation of the Mitochondrial Fission-Fusion Machinery

Drp1 and Marf are well conserved key regulators of mitochondrial dynamics. Hence, we checked whether depleting Drp1 or Marf from dome + ve progenitor subsets (*domeGal4*/+; *UAS mito-GFP*/+; +/+) may affect mitochondrial architecture similar to *asrij* depletion. In anterior lymph gland lobes, mitochondrial branch length increased on Drp1 knockdown (*domeGal4*/+; *UAS mito-GFP*/+; *UAS Drp1 RNAi*/+) indicating mitochondrial fission was inhibited (Figures 1A,C,E). Conversely *Marf* KD (*domeGal4*/+; *UAS mito-GFP*/+; *UAS Marf RNAi*/+) caused mitochondrial fragmentation (reduced mitochondrial branch length) along with reduced mitochondrial content (mitochondrial footprint, number of branches and junctions) indicating reduced fusion (Figures 1A,D,E). Thus, as expected, Drp1 and Marf affect mitochondrial dynamics of blood progenitors. However, there was no significant change in mitochondrial morphology in posterior lobes upon Drp1 depletion (Figures 1A,C,E). *Marf* knockdown reduced mitochondrial branch length in secondary lobe progenitors as compared to control whereas tertiary lobe remained unaffected (Figures 1A,D,E). This suggests that posterior progenitors are less sensitive to perturbation in the mitochondrial fission-fusion machinery.

### Asrij/OCIAD1 Depletion Reduces Mitochondrial Network Dynamics

**FIGURE 2:**
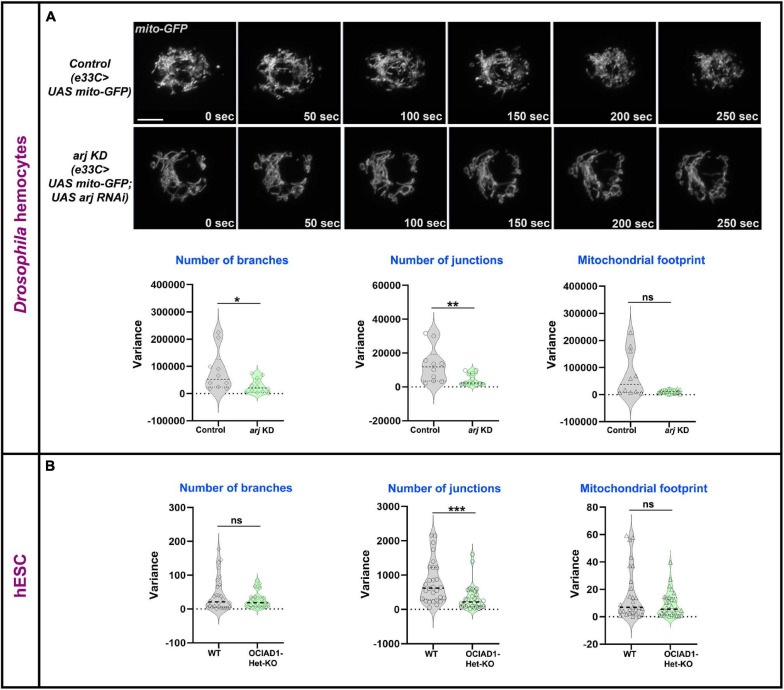
Asrij/OCIAD1 depletion reduces mitochondrial network dynamics. **(A)** Time lapse live imaging of control (*e33CGal* > *UAS mito-GFP*) and *asrij* KD (*e33CGal4* > *UAS mito-GFP; UAS arj RNAi*) circulatory hemocytes expressing mitochondria targeted GFP. Violin plots show quantification of variance in number of branches, number of junctions and mitochondrial footprint in control (*n* = 10 cells) and *arj* KD (*n* = 12 cells) hemocytes. **(B)** Similar quantifications are represented for Mitotracker stained WT (BJNhem20) (*n* = 30 cells) and OCIAD1-Het-KO (CRISPR-39) (*n* = 30 cells) live hESCs. Original data were used from Shetty et al. (2018) for analysis. Scale bar: 5 μm. Error bars represent SEM. Mann-Whitney two-tailed *t*-test was used to determine statistical significance. *P < 0.05, **P < 0.01, ***P < 0.001, ns: statistically non-significant difference.

Mitochondrial dynamics is essential for exchange and distribution of metabolites across the network to different parts of the cell and depends on morphology, number, and branching (Detmer and Chan, 2007). Change of mitochondrial network parameters upon Asrij modulation suggests a possible impact on mitochondrial dynamics. Live imaging analysis of mito-GFP expressing hemocytes from control (*e33C* > *UAS mito-GFP*) and Asrij depleted (Knockdown: KD) (*e33C* > *UAS mito-GFP* > *UAS arj RNAi*) larvae showed lower temporal variation in branch number and junction number with unchanged dynamics of the mitochondrial footprint in KD hemocytes (Figure 2A; Supplementary Video 1). This suggests a possible reduction of mitochondrial fission-fusion events in KD hemocytes and might explain the shift of equilibrium toward elongated mitochondria. Mitochondrial footprint dynamics are unaltered suggesting that mitochondrial biogenesis and degradation may be unaffected, which merits further investigation.

We performed similar analyses in pluripotent human embryonic stem cells (hESCs) that were depleted of OCIAD1 [heterozygous KO (Het-KO): Shetty et al. (2018)]. While the dynamics of branch number and footprint were unchanged, mitochondrial junctions in Het-KO hESCs showed reduced dynamics (Figure 2B). This indicates reduced temporal variation of mitochondrial fission-fusion events upon OCIAD1 depletion in hESCs. As reported earlier, OCIAD1 overexpression in hESCs led to reduction in branch length and mitochondrial footprint (Shetty et al., 2018). Time-lapse image analysis of OCIAD1 overexpressing hESCs showed significantly reduced temporal variation of mitochondrial junction number as compared to control, whereas mitochondrial branch number and footprint dynamics were similar (Supplementary Figure 3). Thus, OCIAD1 depletion and overexpression, both impact mitochondrial dynamics. In summary, modulation of mitochondrial dynamics by Asrij/OCIAD1 is a mechanism that operates in diverse systems such as *Drosophila* blood progenitors and human embryonic stem cells.

### Inhibition of Mitochondrial Fission Prevents Crystal Cell Differentiation

Asrij is essential for lymph gland progenitor maintenance and we find that Asrij depletion alters mitochondrial dynamics. This suggests a possible role for mitochondrial dynamics in progenitor differentiation. Regulated mitochondrial fission and fusion are critical to control mitochondrial dynamics. However, the role of canonical fission and fusion regulators such as Drp1 and Mitofusin in stem cell maintenance and lineage choice is not completely understood.

**FIGURE 3:**
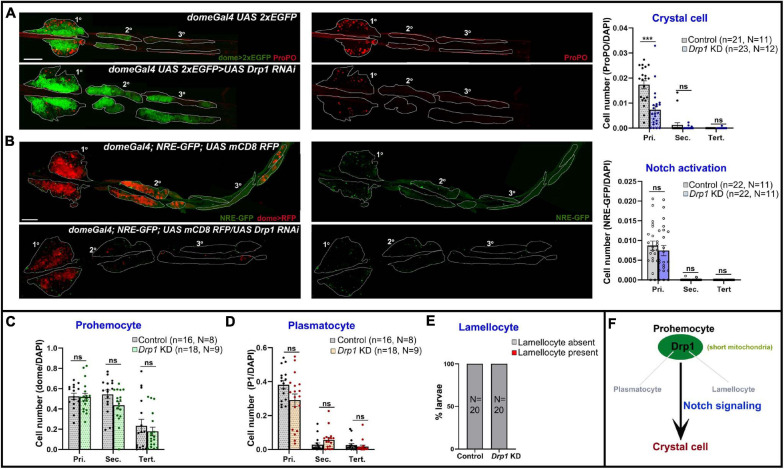
Drp1 regulates blood cell homeostasis in *Drosophila* lymph gland. **(A)** Whole mount lymph gland showing expression of crystal cell marker ProPO in primary, secondary, and tertiary lobes of control (*domeGal4 UAS 2xEGFP*) and *Drp1* KD (*domeGal4 UAS 2xEGFP* > *UAS Drp1 RNAi*) larvae. GFP marks the expression of prohemocyte marker Domeless. Bar diagram shows quantification of mean crystal cell fraction in primary, secondary, and tertiary lobes of indicated genotypes. **(B)** NRE-GFP (Notch responsive element-GFP) reports activation of Notch signaling in control (*domeGal4*/ + ; *NRE-GFP*/ + ; *UAS mCD8 RFP*/ +) and *Drp1* KD (*domeGal4*/ + ; *NRE-GFP*/ + ; *UAS mCD8 RFP/UAS Drp1 RNAi*) lymph gland primary, secondary, and tertiary lobes. RFP marks the expression of prohemocyte marker Domeless. Bar diagram shows quantification of mean NRE-GFP positive (high) cell fraction in primary, secondary, and tertiary lobes of indicated genotypes. **(C–E)** Bar diagrams show quantification of mean dome > 2xEGFP positive prohemocyte fraction **(C)**, P1 positive plasmatocyte fraction **(D)** and percentage of lymph glands with lamellocyte differentiation **(E)** in control and upon Drp1 KD. **(F)** Schematic summarizes effect of Drp1 on various hemocyte lineages and Notch signaling. n represents number of individual lymph gland lobes analyzed, and N represents number of larvae for each genotype. Scale bar: 100 μm. Error bars represent SEM. Multiple *t*-test was performed to determine statistical significance. ***P < 0.001, ns: statistically non-significant difference.

Drp1 drives mitochondrial dynamics by promoting fission and has a role in regulating myeloid reconstitution potential of HSCs (Hinge et al., 2020). However, the role of mitochondrial fission in hematopoietic lineage choice remains largely underexplored. Hence, we examined the effects of depletion of Drp1 from lymph gland progenitors. RNAi-mediated knockdown (KD) in domeless^+^ progenitors (*domeGal4 UAS 2xEGFP;; UAS Drp1 RNAi*) led to reduction in crystal cell (ProPO^+^) differentiation in primary lobes (Figure 3A).

Previous reports show Notch signaling activation is a key mechanism that triggers crystal cell differentiation in the lymph gland while inhibiting differentiation to plasmatocytes or lamellocytes (Duvic et al., 2002; Lebestky et al., 2003; Small et al., 2014; Blanco-Obregon et al., 2020; Cho et al., 2020). However, whether mitochondrial dynamics actively regulate Notch signaling in the lymph gland remains unexplored. We used NRE-GFP (Notch responsive element) reporter to assess the extent of Notch activation upon Drp1 depletion. NRE-GFP (Notch responsive element-GFP) is a widely used reporter for Notch signaling activation. Notch-dependent activation of transcription through NRE promotes GFP transcription. Thus, increased GFP expression marks enhanced activation of Notch signaling. NRE-GFP positive cells are fewer in number in control (*domeGal4*/+; *NRE-GFP*/+; *UAS mCD8 RFP*/+) lymph glands and its expression does not overlap with Dome-positive area or medullary zone (MZ) (Supplementary Figure 4A). Also, it overlaps with the standard crystal cell marker ProPO, indicating active Notch signaling in such cells (Supplementary Figure 4A arrowhead). Progenitor-specific knockdown of *Drp1 (domeGal4*/+; *NRE-GFP*/+; *UAS mCD8 RFP/UAS Drp1 RNAi)* did not affect Notch activation significantly, though there was a downward trend (Figure 3B). This suggests that reduced differentiation to ProPO^+^ crystal cells in *Drp1* KD lymph gland primary lobes may be influenced by other mechanisms downstream of mitochondrial fission in addition to activation of Notch signaling.

Drp1 knockdown had no significant effect on progenitor maintenance, plasmatocyte or lamellocyte differentiation in the lymph gland (Supplementary Figures 5A,B; Figures 3C–E). As depletion of Drp1 affects crystal cell differentiation in primary lobes, we next checked if overexpressing Drp1 had any effect. Progenitor-specific overexpression of Drp1 did not affect crystal cell differentiation (Supplementary Figures 6A,B). Our data suggest that Drp1 selectively regulates crystal cell differentiation (Figure 3F).

### Reduced Mitochondrial Fusion Promotes Notch Signaling and Crystal Cell Differentiation

**FIGURE 4:**
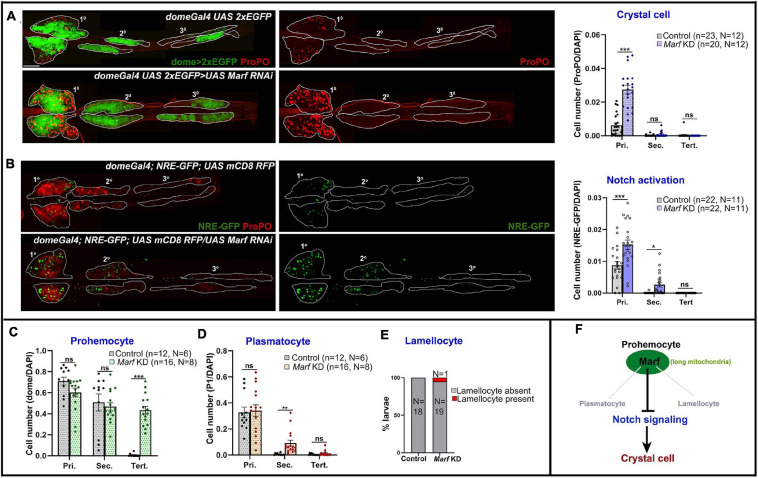
Marf regulates blood cell homeostasis and Notch signaling in *Drosophila* lymph gland. **(A)** Whole mount lymph gland showing expression of crystal cell marker ProPO in primary, secondary, and tertiary lobes of control (*domeGal4 UAS 2xEGFP*) and *Marf* KD (*domeGal4 UAS 2xEGFP* > *UAS Marf RNAi*) larvae. GFP marks the expression of prohemocyte marker Domeless. Bar diagram shows quantification of mean crystal cell fraction in primary, secondary, and tertiary lobes of indicated genotypes. **(B)** NRE-GFP reports activation of Notch signaling in control (*domeGal4*/ + ; *NRE-GFP*/ + ; *UAS mCD8 RFP*/ +) and *Marf* KD (*domeGal4*/ + ; *NRE-GFP*/ + ; *UAS mCD8 RFP/UAS Marf RNAi*) lymph gland primary, secondary, and tertiary lobes. RFP marks the expression of prohemocyte marker Domeless. Bar diagram shows quantification of mean NRE-GFP positive (high) cell fraction in primary, secondary, and tertiary lobes of indicated genotypes. **(C–E)** Bar diagrams show quantification of mean dome > 2xEGFP positive prohemocyte fraction **(C)**, P1 positive plasmatocyte fraction **(D)** and percentage of lymph glands with lamellocyte differentiation **(E)** in control and upon *Marf* KD. **(F)** Schematic summarizes effect of Marf on various hemocyte lineages and Notch signaling. n represents number of individual lymph gland lobes analyzed, and N represents number of larvae for each genotype. Scale bar: 100 μm. Error bars represent SEM. Multiple *t*-test was performed to determine statistical significance. *P < 0.05, **P < 0.01, ***P < 0.001, ns: statistically non-significant difference.

Mitofusins drive mitochondrial dynamics by promoting mitochondrial fusion. Mfn2 regulates maintenance of HSCs with lymphoid potential in mouse through regulation of calcium signaling (Luchsinger et al., 2016). However, its role in myeloid lineage specification is not fully understood. Knockdown of *Drosophila* Mfn homolog Marf (*domeGal4 UAS 2xEGFP;; UAS Marf RNAi*) led to dramatic increase in crystal cell differentiation in primary lobes (Figure 4A). Both Drp1 and Marf play critical but opposite roles in non-canonical Notch signaling activation and various developmental processes such as neuroblast and synaptic development in *Drosophila* (Lee et al., 2013; Sandoval et al., 2014). In concordance with previous reports, we find that *Marf* KD *(domeGal4*/ + ; *NRE-GFP*/ + ; *UAS mCD8 RFP/UAS Marf RNAi)* led to increase in Notch activation in primary and secondary lobes (Figure 4B) whereas tertiary lobes remained unaffected. This explains increased crystal cell differentiation in *Marf* KD lymph gland primary lobes. Absence of crystal cells in the tertiary lobes suggests additional regulatory mechanisms and is in agreement with the idea that posterior lobes resist differentiation (Rodrigues et al., 2021).

**FIGURE 5:**
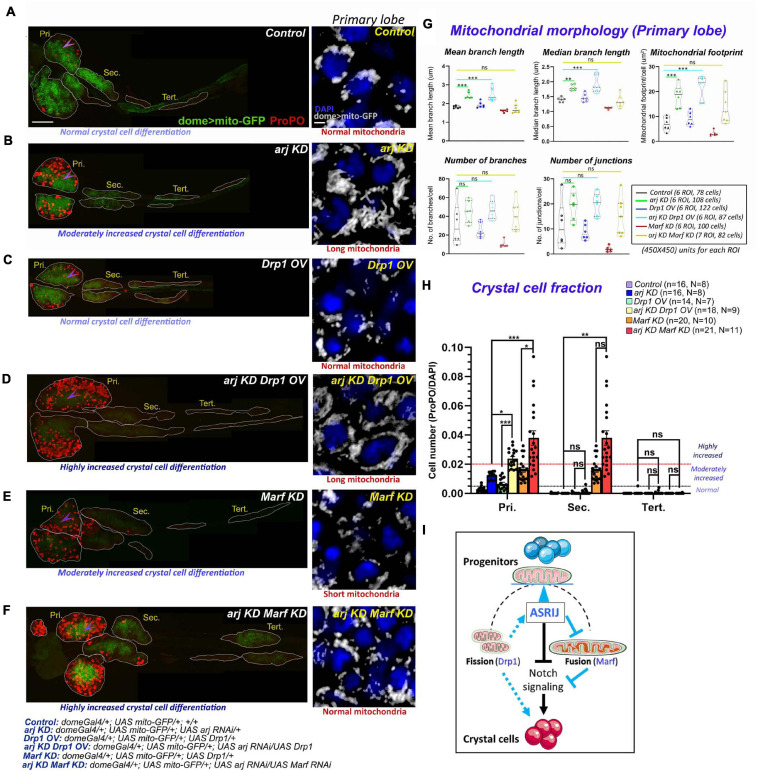
Progenitor-specific genetic interaction of *asrij* with *Drp1* and *Marf* controls crystal cell differentiation in the lymph gland. **(A–F)** Whole mount lymph gland showing ProPO expression (far red pseudo-colored to red) to mark crystal cells in primary (Pri.), secondary (Sec.), and tertiary (Tert.) lobes of *control* **(A)**, *arj KD* **(B)**, *Drp1 OV* **(C)**, *arj KD Drp1 OV* **(D)**, *Marf KD* **(E)**, and *arj KD Marf KD* **(F)** larvae. The phenotypes of crystal cell differentiation are mentioned below each lymph gland image. The detailed genotypes are mentioned below the images panel for lymph gland. Scale bar: 100 μm. Mitochondrial morphology (dome > mito-GFP expression) in the primary lobe progenitors (marked by arrowhead) is shown adjacent to the lymph gland images of the respective genotypes in gray scale. The phenotype of mitochondria morphology is mentioned below each image. Single confocal slice of 0.5 μm is represented for easy visualization of mitochondrial network. Scale bar: 5 μm. **(G,H)** Mitochondrial morphology analysis is shown for the abovementioned genotypes **(G)**. Bar diagrams show quantification of ProPO positive cell fraction in different lobes of the same genotypes **(H)**. Error bars represent SEM. The values have been classified as normal (0–0.005), moderately increased (0.005–0.02) and highly increased (>0.02). n represents number of individual lymph gland lobes analyzed, and N represents number of larvae for each genotype. One-way ANOVA was performed to determine statistical significance for mito-GFP quantitation while Kruskal-Wallis test was performed for analysis of crystal cell fraction. *P < 0.05, **P < 0.01, ***P < 0.001, ns: non-significant. **(I)** Schematic representation of the effect of mitochondrial morphology and dynamics on blood cell differentiation. Asrij is a hub that maintains the balance (blue arrowhead) between mitochondrial fission and fusion to regulate progenitor maintenance and crystal cell differentiation. Arrows indicate activation. T symbol indicates inhibition. Black color indicates previously known interactions; blue color indicates effects reported in this study.

*Marf* knockdown did not affect the primary and secondary lobe progenitors. However, surprisingly, the dome + progenitor fraction increased in tertiary lobes (Supplementary Figure 5C; Figure 4C). This could be due to increased proliferation of posterior progenitors that have inherently reduced differentiation potential. Plasmatocyte differentiation remained unchanged in primary and tertiary lobes, increasing mildly in secondary lobes (Supplementary Figure 5C; Figure 4D). Occasionally there was a small increase (1 out of 20 larvae analyzed) in lamellocyte differentiation (Supplementary Figure 5D; Figure 4E). This indicates that Marf activity prevents dome^+^ progenitor expansion in the posterior lobes and primarily prevents precocious crystal cell differentiation (Figure 4F). Marf overexpression increased crystal cell differentiation in primary lobes. However, posterior lobes were unaffected (Supplementary Figures 6A,C). This indicates differences in progenitor sensitivity to Marf levels.

Taken together our analyses show that canonical mitochondrial dynamics regulators such as Drp1 and Marf actively modulate Notch activation to dictate crystal cell differentiation in the *Drosophila* lymph gland.

### Asrij Integrates Mitochondrial Dynamics With Crystal Cell Differentiation

Loss of Asrij leads to enhanced activation of Notch signaling with a concomitant increase in crystal cell differentiation (Supplementary Figure 4B) (Kulkarni et al., 2011; Khadilkar et al., 2014). Since Asrij also regulates mitochondrial dynamics, we next asked whether Asrij genetically interacts with the canonical regulators of mitochondrial dynamics. Elongated mitochondria in *asrij* KD progenitors could be a result of impaired fission or enhanced fusion events and hence are expected to be rescued by promoting fission (Drp1 overexpression) or inhibiting fusion (Marf depletion).

Progenitor-specific Drp1 overexpression (OV) using *domeGal4* driver could not efficiently restore normal mitochondrial architecture in Asrij depleted progenitors (*arj KD Drp1 OV: domeGal4*/ + ; *UAS mito-GFP*/ + ; *UAS Drp1/UAS arj RNAi*) (Figures 5A–D insets and Figure 5G). However, Marf knockdown (*Marf KD*), which caused fragmentation of mitochondria rescued *asrij* KD phenotype in progenitors (*arj KD Marf KD: domeGal4*/ + ; *UAS mito-GFP*/ + ; *UAS Marf RNAi/UAS arj RNAi*) (Figures 5A,B,E,F insets and Figure 5G). This suggests that elongation of mitochondria in *asrij* KD condition is an outcome of enhanced fusion rather than impaired fission.

We also analyzed the extent of crystal cell differentiation in the lymph glands of these genotypes. Increased Drp1 (OV) or reduced Marf (KD) in progenitors in the *asrij* depleted background (*arj KD*) showed a synergistic effect on the crystal cell phenotype. Both *arj* KD *Drp1* OV and *arj* KD *Marf* KD lymph gland primary lobes showed a greater increase in crystal cells compared to single mutants *arj* KD, *Drp*1 OV or *Marf* KD (Figures 5A–F,H). There was no significant increase in crystal cell differentiation in posterior lobes except in the secondary lobes of *arj* KD *Marf* KD compared to control or *arj* KD. As Drp1 overexpression could not rescue mitochondrial elongation caused by loss of Asrij, it may function upstream of Asrij to regulate mitochondrial phenotype. However, increased crystal cell differentiation upon Drp overexpression, which is enhanced in the *asrij* mutant background, suggests a direct effect on crystal cell differentiation. On the other hand, Marf acts downstream of Asrij in blood progenitors to regulate mitochondrial dynamics and crystal cell differentiation (Figure 5I). Deciphering how these regulators of mitochondrial architecture control lineage-specific progenitor differentiation requires further investigation.

Similar results were observed using the pan-hemocyte driver e33CGal4. Drp1 overexpression or Marf KD in *asrij* null hemocytes *(arj9/arj9; e33CGal4/UAS Drp1 and arj9/arj9; e33CGal4/UAS Marf RNAi)* rescued normal mitochondrial architecture (branch length and aggregation), comparable to control (*w1118*) (Supplementary Figure 7). However, although Marf KD rescued increased mitochondrial footprint (content) in *asrij* null hemocytes, Drp1 overexpression could not. This suggests inefficient clearance of mitochondria even after Drp1 overexpression. Also, it reaffirms our claim that elongation of mitochondria on Asrij depletion is mostly due to enhanced mitochondrial fusion rather than decreased fission. Crystal cell differentiation in the primary lobe increased in a synergistic manner upon Drp1 OV or Marf KD in *asrij* null lymph gland (Supplementary Figure 8). Hence, Drp1 overexpression and Marf depletion affect crystal cell differentiation in similar way upon loss of Asrij. This suggests Asrij depletion makes cells more susceptible to the effect of increasing fission or reducing fusion implying greater sensitivity to mitochondrial dynamics. Hence, distinct functional networks connecting Asrij to the fission-fusion machinery may maintain normal mitochondrial dynamics and optimum differentiation of crystal cells.

## Discussion

Mitochondria play an indispensable role in cell fate choice both in healthy tissue and in diseased conditions (Zhang et al., 2018). Mitochondrial dysfunction underlies several cytopathological conditions including neurological disorders and cancers (Annesley and Fisher, 2019). Perturbed mitochondrial function in stem cells may affect their proliferation or differentiation (Seo et al., 2018). Recently genetic models have been used to validate the role of mitochondria in hematopoietic stem cell maintenance and differentiation (Diebold and Chandel, 2016; Filippi and Ghaffari, 2019). Here we combine analysis of an invertebrate *in vivo* model with an *in vitro* human stem cell model to understand the role of mitochondrial dynamics and its regulators in lineage specification.

Given the lack of information about mitochondrial morphology in *Drosophila* hematopoietic progenitors, we first undertook a detailed mapping in these cells. To fully exploit the power of the lymph gland model, we chose to analyze the progenitor population in all lobes of the lymph gland as these represent temporally distinct stages of progenitor maturation and differing propensity for differentiation. We found differences in mitochondrial morphology between more mature anterior progenitors and younger posterior progenitors. Further, analysis of the effect of modulating mitochondrial fission-fusion regulators as well as Asrij also showed different responses in progenitor subsets. Our results are in agreement with the idea that posterior progenitors differ from anterior ones in their identity and function. The physiological relevance of this appears to be context dependent – for example, Asrij-dependent mitochondrial phenotypes affect progenitor fate choice in the lymph gland but may have additional roles in differentiated hemocytes that merit further investigation. Our detailed studies position the *Drosophila* lymph gland as a relevant and accessible *in vivo* model to study mitochondrial regulation of progenitor heterogeneity that is not currently possible in vertebrate models. It would be interesting to see whether expression or activity of OCIAD1 within vertebrate stem cell pools can contribute to heterogeneity and fate choice through regulation of mitochondrial function.

Mitochondrial morphology is inextricably related to its function including oxidative phosphorylation (Wai and Langer, 2016). Therefore, mitochondrial dynamics could serve as a potential therapeutic target for several diseases with mitochondrial dysfunction (Brandner et al., 2019; Whitley et al., 2019). While the role of Drp1 and Mfn2 in mitochondrial dynamics is well established, their ability to regulate hematopoiesis in vertebrates has been reported only recently (Luchsinger et al., 2016; Hinge et al., 2020). Misexpression of DNM1L and Mfn1/2 may underlie several human hematological malignancies including acute myeloid leukemia (AML), chronic myeloid leukemia (CML), chronic lymphocytic leukemia (CLL) and myelodysplastic syndromes^1^ ^,2^. However, these ubiquitous regulators of mitochondrial dynamics are not suitable therapeutic targets. Therefore, identification of tissue-restricted regulators of mitochondrial dynamics that are not essential for viability, is important.

Asrij, a pan-hematopoietic protein regulates hematopoiesis (Inamdar, 2003; Kulkarni et al., 2011), yet complete loss of Asrij does not cause lethality in *Drosophila* (Kulkarni et al., 2011) or in mouse (Sinha et al., 2019a). Hence, *asrij* null (*arj9/arj9*) flies serve as an excellent *in vivo* model for leukemia and hematopoietic anomalies. OCIAD1 has been implicated in several pathological conditions including ovarian carcinoma, myelodysplastic syndromes, and mitochondrial disorders (Praveen et al., 2020). Here using cross-species comparison and *in vivo* analysis, we show a conserved role for Asrij/OCIAD1 in regulating cell fate through mitochondrial dynamics. Asrij has an indispensable role in specifying myeloid biased fate of blood progenitors (Kulkarni et al., 2011; Sinha et al., 2019a). Asrij-dependent mitochondrial dynamics is a potential mechanism to regulate myeloid specification. Further we identify Asrij as a common modulator of mitochondrial fission and fusion that controls Notch signaling for crystal cell differentiation.

Asrij depletion causes elongation of mitochondria that could be rescued by suppressing mitochondrial fusion. OCIAD1 inhibits Complex I activity and oxygen consumption to suppress excessive early mesodermal progenitor formation from hESCs (Shetty et al., 2018). Hence, change in mitochondrial dynamics upon depletion or overexpression of OCIAD1 could possibly underpin its impact on respiration and downstream metabolic or signaling pathways.

We show that fission and fusion regulators Drp1 and Marf act through distinct networks to effect differentiation. Though Drp1 and Mfn2 play critical roles in mouse hematopoiesis, their role in lineage-specific signaling activation is unclear. Using the *Drosophila* lymph gland as an *in vivo* model of hematopoiesis, we find critical roles for canonical mitochondrial dynamics regulators such as Drp1 and Marf (dMfn) in blood cell differentiation. Drp1 and Marf play opposite roles in Notch activation for progenitor differentiation to crystal cells. Marf inhibits Notch activation and crystal cell differentiation whereas Drp1 may promote it. This is in concordance with previous reports showing opposing effects of Drp1 and Marf on developmental processes such as neuroblast and synaptic development in *Drosophila* larva (Mitra et al., 2012; Lee et al., 2013; Sandoval et al., 2014). Moreover, it supports a previously reported positive role of Drp1 in Notch activation in *Drosophila* germline and neural stem cells (Mitra et al., 2012; Lee et al., 2013). Hence, antagonistic roles of Drp1 and Marf in mitochondrial dynamics may mediate balanced activation of Notch signaling and lineage-specific differentiation of lymph gland progenitors.

Drp1 feeds back to activate Notch signaling in triple negative breast cancer cells which in turn can upregulate Drp1-dependent mitochondrial fission (Chen et al., 2018). On the other hand, we find that Marf KD promotes Notch activation. Hence, mitochondrial morphology/dynamics and Notch signaling can regulate each other. Further, Notch activation should result in fragmented mitochondria. Though Notch is activated on Asrij depletion (Kulkarni et al., 2011), mitochondria are elongated and this phenotype is rescued by Marf KD, indicating that Asrij acts upstream to enhance Notch activation, which is in agreement with our earlier report. However, additional regulatory mechanisms may also be in play. Nevertheless, we show that a functional network of Asrij with canonical mitochondrial dynamics regulators (Drp1 and Marf) synergistically regulates crystal cell differentiation, a lineage downstream to Notch signaling. Mfn2 and DNM1L (Drp1) are reported as components of a proximity interaction network of OCIAD1, thus supporting further our claim of a direct functional interaction of Asrij with these canonical regulators of mitochondrial dynamics (Antonicka et al., 2020)^3^. Hence, Asrij-dependent Notch signaling may possibly lie downstream of the Asrij-dependent mitochondrial dynamics.

Despite extensive studies on the role of mitochondria in vertebrate hematopoiesis, their role in regulating lymph gland lineage choice remains elusive. ROS prime progenitors for differentiation to all hemocyte lineages (Owusu-Ansah and Banerjee, 2009). Even though ROS levels are susceptible to change with shift in mitochondrial dynamics (Bhandari et al., 2015; Senos Demarco and Jones, 2019), our results show that the impact of Drp1 or Marf depletion is limited to Notch pathway activation and crystal cell differentiation. This suggests additional mechanisms that may make plasmatocyte and lamellocyte differentiation sensitive to inhibition of mitochondrial fission or fusion.

It is quite possible that Asrij and Marf may have similar roles in some other aspects of mitochondrial function that regulate Notch activation. Probably that is why the combined knockdown of Asrij and Marf, although rescues mitochondrial morphology, cannot rescue increased crystal cell differentiation but rather enhances it synergistically. Previous studies have reported physical interaction of OCIAD1 with regulators of calcium signaling that depends on ER-mitochondria interaction (Floyd et al., 2016). This raises the possibility that other inter-organelle mechanisms may also be involved.

Posterior subsets of lymph gland progenitors resist differentiation upon infection as they are younger and developmentally less mature as compared to primary lobe progenitors. However, mechanisms that control posterior progenitor fate are only recently being understood (Krzemien et al., 2010; Rodrigues et al., 2021). Our results show progenitor-specific Marf depletion causes mild increase in plasmatocyte differentiation in secondary lobes suggesting mitochondrial fragmentation as a possible mechanism to trigger differentiation in posterior subsets of progenitors. Also, tertiary lobe progenitor population increases upon Marf depletion. This could be a basis for screens to identify reversal of such phenotypes and lead to novel position-specific regulators of progenitors.

Mitochondrial metabolism and dynamics are inter-dependent (Wai and Langer, 2016). We show a conserved role for Asrij in both mitochondrial morphology, dynamics, and function. Hence, Asrij/OCIAD1 may be a key conserved regulator that coordinates different facets of mitochondrial activity, to dictate cell fate decisions. We observed increased mitochondrial content in Asrij depleted hemocytes. This could be due to impaired mitophagy that is often seen upon reduced fission or hyperfusion of the mitochondrial network (Twig et al., 2008). Although Marf depletion can reduce the mitochondrial content in Asrij depleted cells, Drp1 overexpression fails to do so suggesting that Drp1 cannot sufficiently promote mitochondria clearance, possibly through mitophagy, in Asrij depleted condition. This also indicates that mitochondrial fusion rather than fission has a pivotal role in Asrij-dependent blood cell homeostasis.

Both mitophagy and mitochondria-derived vesicle biogenesis control mitochondria quality. These pathways establish a functional link between mitochondria and endocytic compartments. Recent reports show a potential role of mitophagy in maintaining hematopoietic progenitors or stimulating hematopoiesis in vertebrates (Jin et al., 2018; Girotra et al., 2020). Such inter-organelle communications in signaling homeostasis and downstream cell fate specification could allow for complex spatial and temporal regulation. While canonical Notch signaling leads to crystal cell differentiation, non-canonical activation of Notch pathway due to stalling in endosomes of Asrij mutants (Kulkarni et al., 2011) may also contribute to the synergistic effects on phenotype. Asrij/OCIAD1 acts as a transmembrane scaffolding protein that regulates assembly and activation of critical signaling components and molecular complexes across organelles (Sinha et al., 2013; Le Vasseur et al., 2021). A recent report shows that OCIAD1 is a client of Prohibitin supramolecular complex that acts as a scaffold for the assembly of mitochondrial electron transport chain Complex III in HEK293T and U2OS cells (Le Vasseur et al., 2021). Hence, Asrij may potentially act as mediator of inter-organellar communication that may influence blood cell homeostasis.

Notch activation increases in secondary lobes upon Marf depletion suggesting that progenitors are primed, but not fully committed toward differentiation. This may also reflect their immature developmental stage and functional heterogeneity. Other mechanisms may operate to inhibit crystal cell differentiation (ProPO^+^) downstream to Notch activation in secondary lobes of Marf depleted lymph glands. Further, progenitor fraction in tertiary lobes increases upon Marf depletion indicating possible increase in progenitor proliferation. On the other hand, Drp1 depletion does not affect blood cell homeostasis in posterior lobes. Hence, Marf-dependent mitochondrial dynamics could be a position-dependent mechanism to regulate posterior progenitors. Further, it raises the possibility that a subset of dome + progenitors are biased toward crystal cell differentiation and that progenitors may differ in lineage potential. Importantly, this effect is position-dependent as dome + posterior progenitors in secondary lobes, even after undergoing mitochondrial fragmentation due to Marf depletion, fail to differentiate, unlike primary lobe progenitors. It also implies that the posterior identity of progenitors is maintained even on perturbing mitochondrial morphology, as they continue to be refractile to differentiation. Asrij depletion may unlock lineage differentiation potential to assist progenitor differentiation in posterior lobes.

Even though we show the effect of progenitor-specific knockdown of *asrij*, *Drp1* and *Marf* on blood cell differentiation, any non-autonomous impact cannot be ruled out. Blood cell differentiation in such cases could be due to differentiation of the KD progenitor itself or due to signals originating from the KD progenitors that promote differentiation or trans-differentiation of intermediate progenitors or differentiated hemocytes. Our results show genetic interaction between Asrij and Drp1/Marf within the same pool of cells - either circulatory hemocytes or lymph gland progenitors. So, it is quite possible that the functional synergy to regulate crystal cell differentiation could be due to genetic interaction in the same cell. However, effect on crystal cell differentiation could be cell non-autonomous as well, which can be tested by mitotic mutant clone analysis.

Our data support an interplay of the blood cell enriched protein Asrij with mitochondrial dynamics regulators Drp1 and Marf in lineage-specific differentiation. Given the ubiquitous requirement for Drp1 and Marf and the pan-hemocyte expression of Asrij, it is quite unexpected to see such lineage specific effects. These insights validate our use of *Drosophila* genetics and the *in vivo* lymph gland hematopoiesis model to uncover such complex and unique interactions. Further they reveal Asrij as a critical regulatory node connecting mitochondrial dynamics, Notch signaling and crystal cell differentiation. Our findings suggest that the functional output of mitochondrial dynamics may be beyond simply the mitochondrial network architecture and depends on other unidentified factors linked to the dynamicity of this network. Modulating mitochondrial dynamics *in vitro* can serve as a way to promote or inhibit lineage-specific differentiation for therapeutic purposes. In summary, Asrij-regulated mitochondrial dynamics emerge as a potential conserved mechanism to maintain blood cell homeostasis.

## Data Availability Statement

The original contributions presented in the study are included in the article/Supplementary Materials, further inquiries can be directed to the corresponding author/s.

## Author Contributions

AR performed the experiments. MI contributed reagents and materials. All authors designed research, analyzed the data, wrote the manuscript, contributed to the article, and approved the submitted version.

## Conflict of Interest

The authors declare that the research was conducted in the absence of any commercial or financial relationships that could be construed as a potential conflict of interest.

## Footnotes

1 http://servers.binf.ku.dk/bloodspot

2 https://cancer.sanger.ac.uk/cosmic

3 https://thebiogrid.org/120280/summary/homo-sapiens/ociad1.html

## Supplementary Material

Supplementary Figure 1 (related to Figure 1) Asrij regulates mitochondrial morphology in *Drosophila* circulatory hemocytes.

Supplementary Figure 2 (related to Figure 2 and Supplementary Figure 1) Asrij overexpression affects mitochondrial network architecture in hemocytes.

Supplementary Figure 3 (related to Figure 2) OCIAD1 overexpression affects mitochondrial dynamics in hESCs.

Supplementary Figure 4 (related to Figures 3–5) NRE-GFP expression increases upon *asrij* knockdown.

Supplementary Figure 5 (related to Figures 3, 4) Drp1 and Marf regulate blood cell homeostasis in *Drosophila* lymph gland.

Supplementary Figure 6 (related to Figures 3, 4) Overexpression of Drp1 and Marf differentially affect crystal cell differentiation in *Drosophila* lymph gland.

Supplementary Figure 7 (related to Figure 5) Genetic interaction of *asrij* with Drp1 and *Marf* controls mitochondrial network architecture in circulatory hemocytes.

Supplementary Figure 8 (related to Figure 5) Pan-hemocyte-specific genetic interaction of *asrij* with *Drp1* and *Marf* controls crystal cell differentiation in lymph gland.

Supplementary Video 1 (related to Figure 2) Asrij regulates mitochondrial dynamics in hemocytes.
