# Supplementary material for: A Conserved Role for Asrij/OCIAD1 in Progenitor Differentiation and Lineage Specification Through Functional Interaction With the Regulators of Mitochondrial Dynamics
Source: Front Cell Dev Biol. 2021 Jul 6;9:643444. doi: 10.3389/fcell.2021.643444 (PMC8290362; doi:10.3389/fcell.2021.643444)
Supplement: Supplementary Figure 1 — (related to Figure 1) Asrij regulates mitochondrial morphology in Drosophila circulatory hemocytes. [file Data_Sheet_1.pdf]

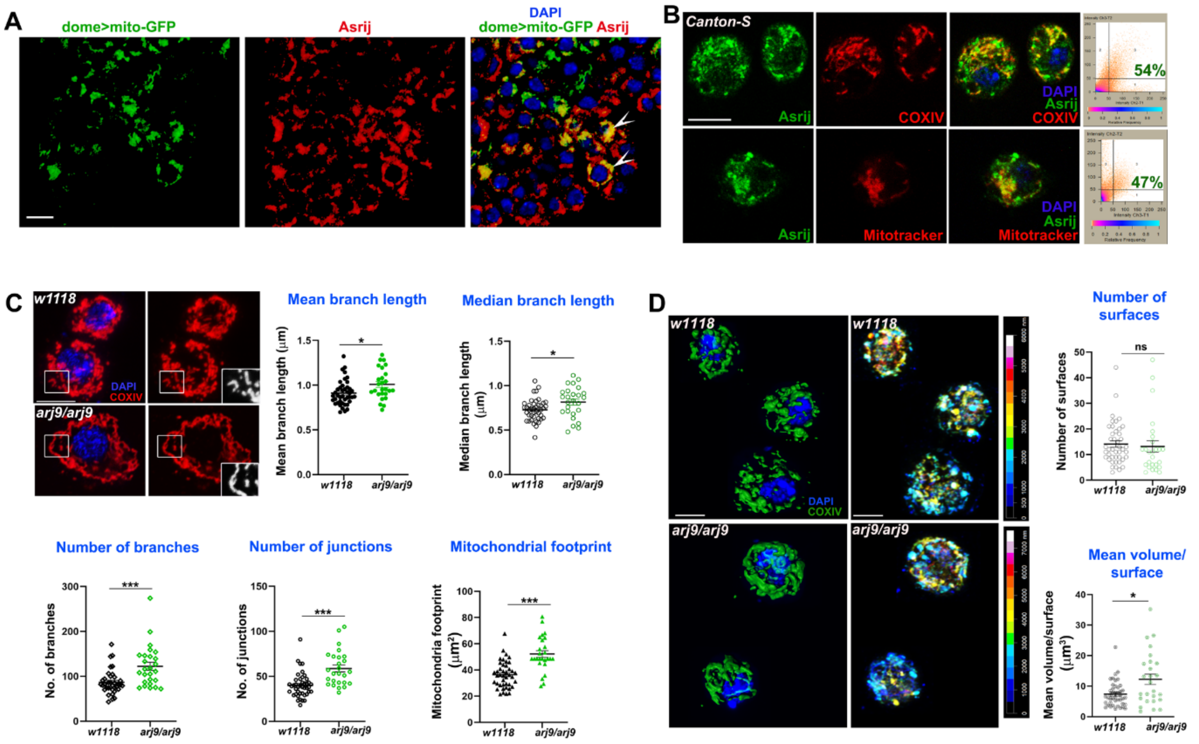

Supplementary Figure S1. Asrij regulates mitochondrial morphology in *Drosophila* circulatory hemocytes.

(A-B) Asrij (far red pseudo-colored to red) colocalization with mitochondrial marker mito-GFP (green) in blood progenitors of the lymph gland (*domeGal4/+; UAS mito-GFP/+;+/+*) (A). Image represents single 0.5 µm confocal slice to show colocalization. Arrowheads mark the site of colocalization. DAPI marks the nuclei. Scale bar: 5 µm. (B) Asrij (green) colocalization with mitochondrial markers (red) COXIV or Mitotracker as indicated in wild type (Canton-S) hemocytes in circulation. Colocalization plots are as indicated for 0.3 µm optical section.

(C) Mitochondria stained by COXIV in control (*w1118*) and *asrij* null (*arj9/arj9*) circulatory hemocytes. Insets (grey scale) show magnified view of boxed region. Scatter plots show quantification of mitochondrial mean branch length, median branch length, number of branches, number of junctions and mitochondrial footprint in *w1118* (n=43 cells) and *arj9/arj9* (n=26 cells) circulatory hemocytes.

(D) Maximum intensity projection images reconstructed in three dimensions (left) and depth color-coded projections (right) for COXIV staining in *w1118* and *arj9/arj9* hemocytes. Three-dimensional surface reconstruction was used to determine number of surfaces and mean volume per surface in each cell, shown in the scatter plot.

Scale bars in all panels barring (A): 10 µm. Error bars represent SEM. Mann-Whitney two-tailed t-test was used to determine statistical significance. *P<0.05, *** P <0.001, ns: statistically non-significant difference.

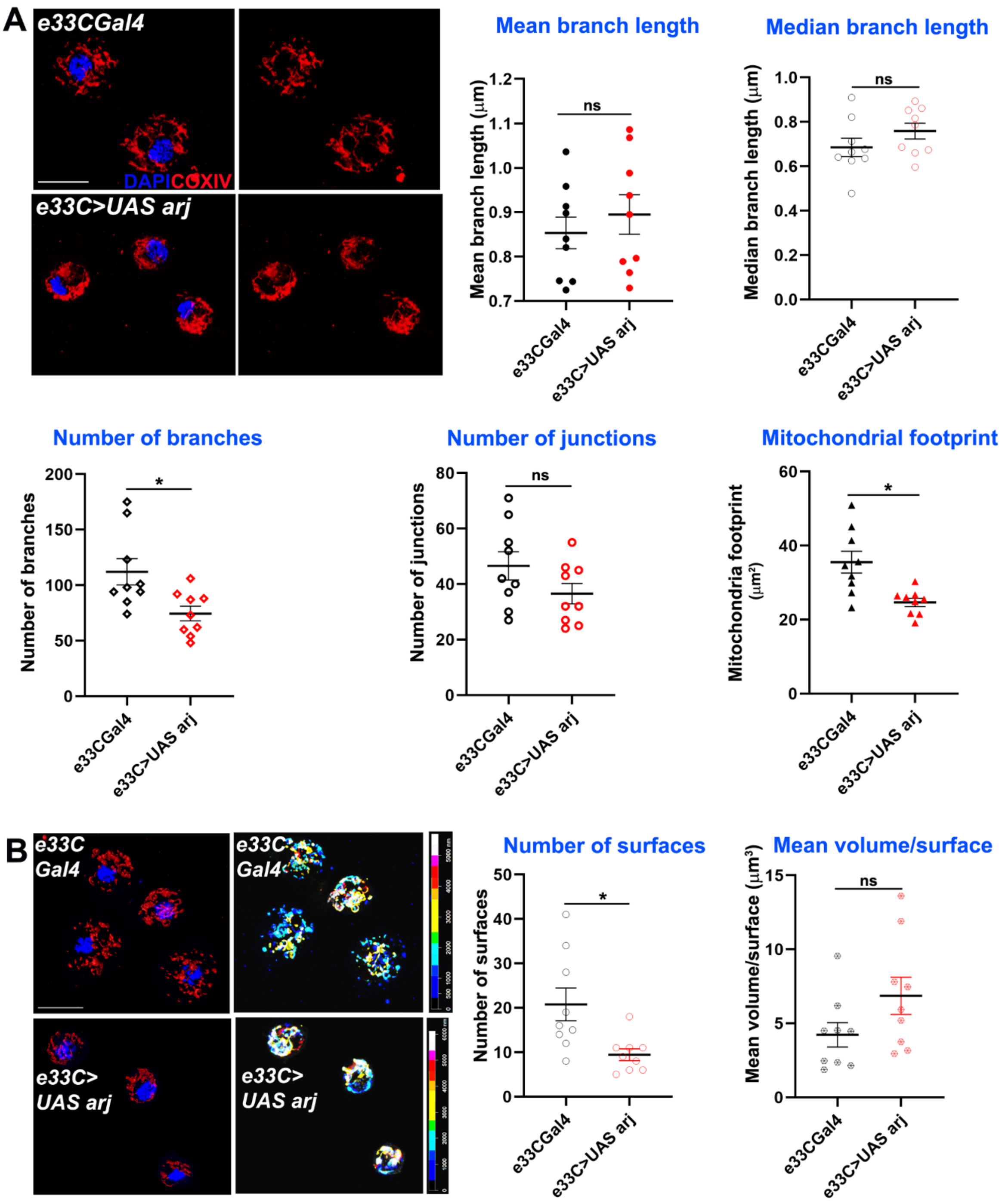

Supplementary Figure S2. Asrij overexpression affects mitochondrial network architecture in hemocytes

(A) COXIV staining marks mitochondria in parental control (*e33CGal*) and Asrij overexpressing (*e33C>UAS arj*) larval circulatory hemocytes. Scatter plots show quantification of mitochondrial mean branch length, median branch length, number of

branches, number of junctions and mitochondrial footprint in *e33CGal4* (n=9 cells) and *e33C>UAS arj* (n=9 cells) circulatory hemocytes.

(B) Maximum intensity projection images were reconstructed in three dimensions for COXIV staining in *e33CGal4* and *e33C>UAS arj* hemocytes. Same images are also represented by depth color-coded projections. COXIV staining three-dimensional surface reconstruction was used to determine number of surfaces and mean volume per surface in each cell.

Scale bars in all panels: 10 μm. Error bars represent SEM. One-way ANOVA was used to determine statistical significance. *P<0.05, ns: statistically non-significant difference.

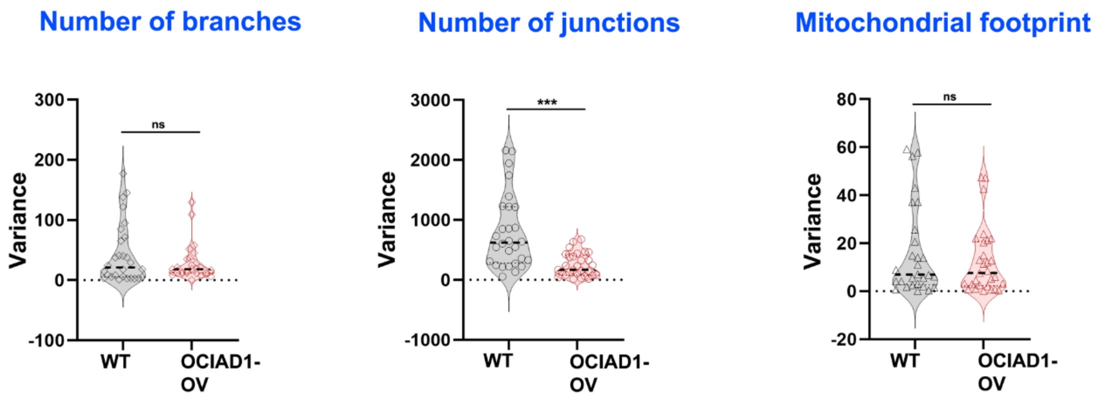

Supplementary Figure S3. OCIAD1 overexpression affects mitochondrial dynamics in hESCs

Violin plots show quantification of variance in number of branches, number of junctions and mitochondrial footprint in mitotracker stained WT (BJNhem20) (n=30 cells) and OCIAD1-OV (n=30 cells) live hESCs. Original data were used from Shetty et al., 2018 for analysis.

Error bars represent SEM. Mann-Whitney two-tailed t-test was used to determine statistical significance. ***P <0.001, ns: statistically non-significant difference.

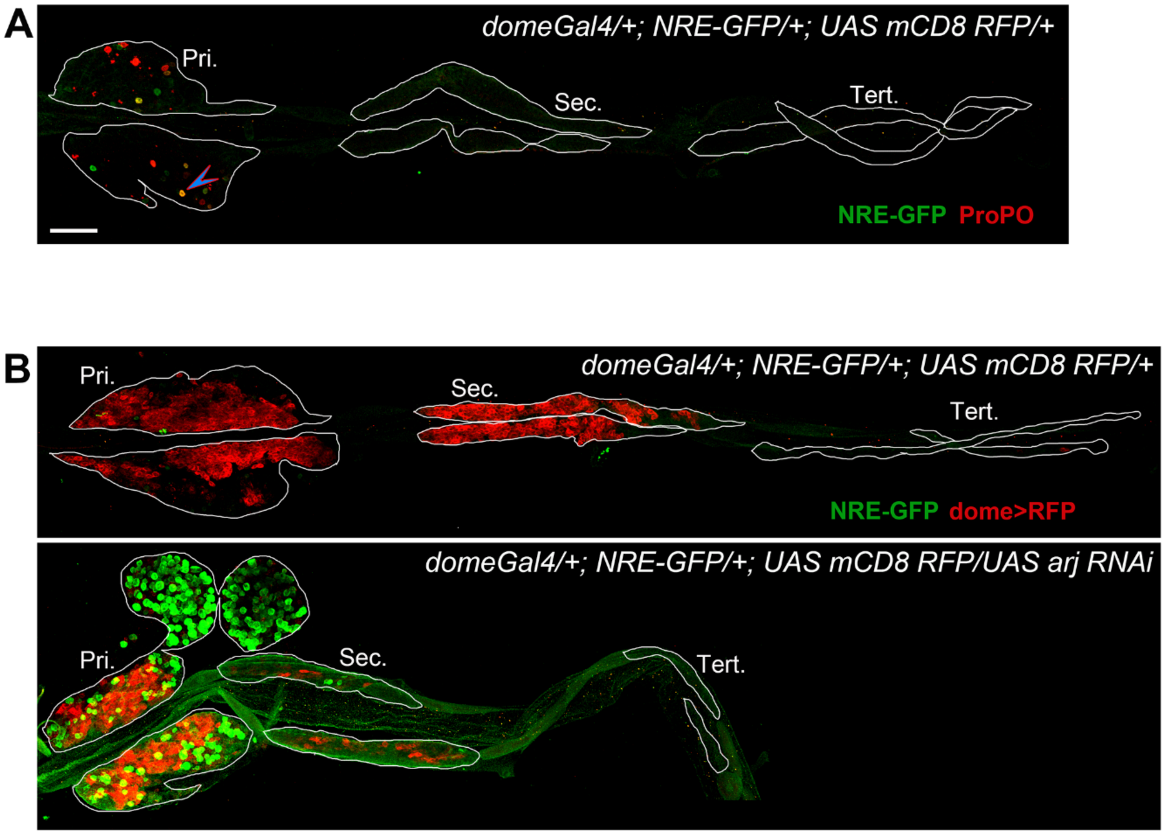

Supplementary Figure S4. NRE-GFP expression increases upon *asrij* knockdown

(A) Notch responsive element-GFP (NRE-GFP) positive cells are present in primary lobes (Pri.) and the expression overlaps with ProPO as marked by arrowhead. Scale bar: 100 µm.

(B) NRE-GFP reporter positive cells mark Notch activation in control (*domeGal4/+; NRE-GFP/+; UAS mCD8 RFP/+*) and *asrij* knockdown (*domeGal4/+; NRE-GFP/+; UAS mCD8 RFP/UAS arj RNAi*) lymph glands. Progenitors are marked by dome>RFP.

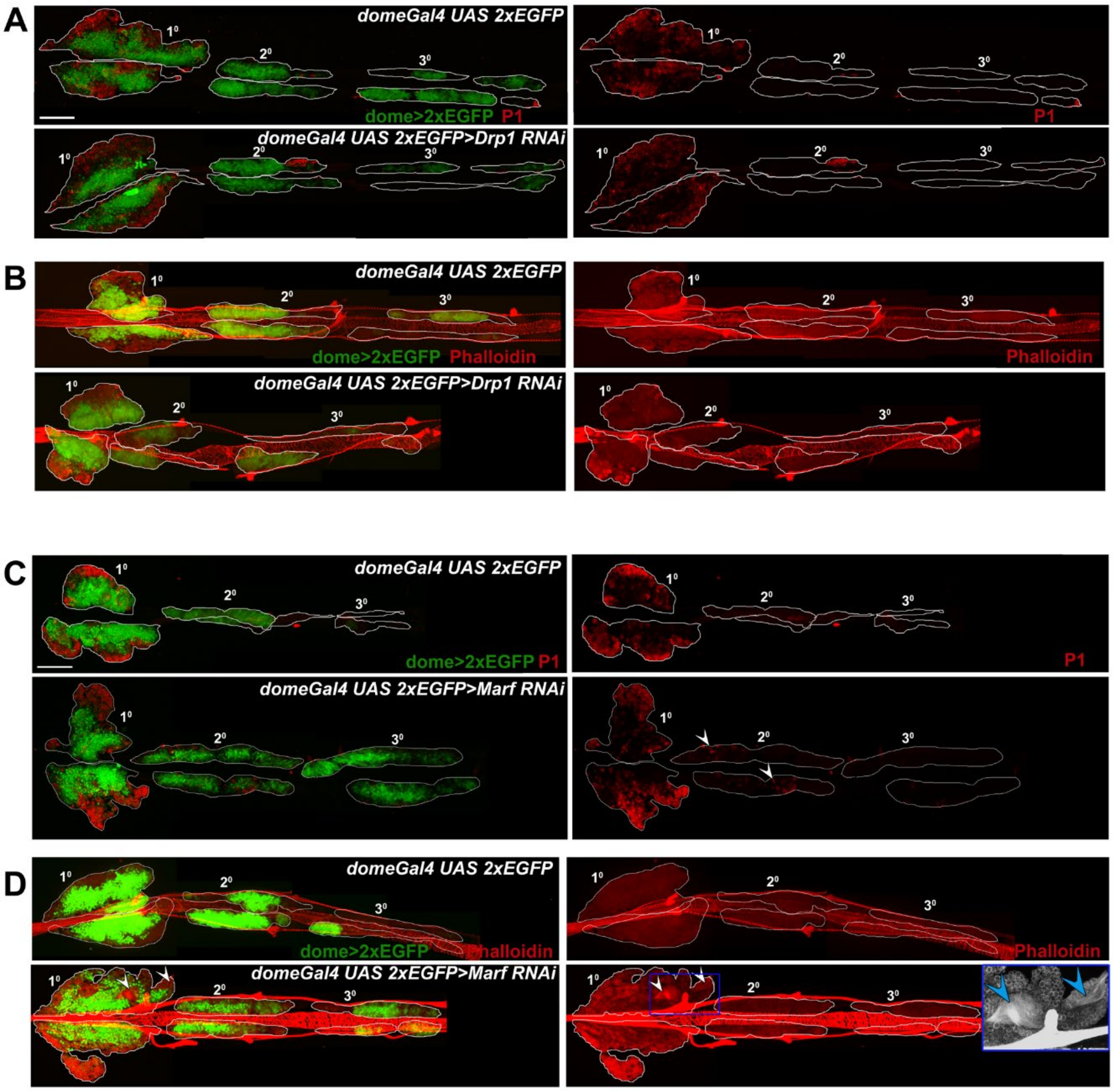

Supplementary Figure S5. Drp1 and Marf regulate blood cell homeostasis in *Drosophila* lymph gland

(A-B) Whole mount lymph gland showing plasmatocytes marker P1 expression in primary, secondary, and tertiary lobes (A). Phalloidin staining shows lamellocytes (B) in control (*domeGal4 UAS 2xEGFP*) and *Drp1* KD (*domeGal4 UAS 2xEGFP>UAS Drp1 RNAi*) lymph gland lobes. dome>2xEGFP marks prohemocytes.

(C-D) P1 staining marks plasmatocytes (C) and Phalloidin staining shows lamellocytes (D) in control (*domeGal4 UAS 2xEGFP*) and Marf KD (*domeGal4 UAS 2xEGFP>UAS Marf RNAi*)

lymph gland lobes. Arrowheads mark plasmatocytes (C) and lamellocytes (D) in *Marf* KD lymph gland. Insets show magnified view of lamellocytes in boxed region.

Scale bar: 100 µm.

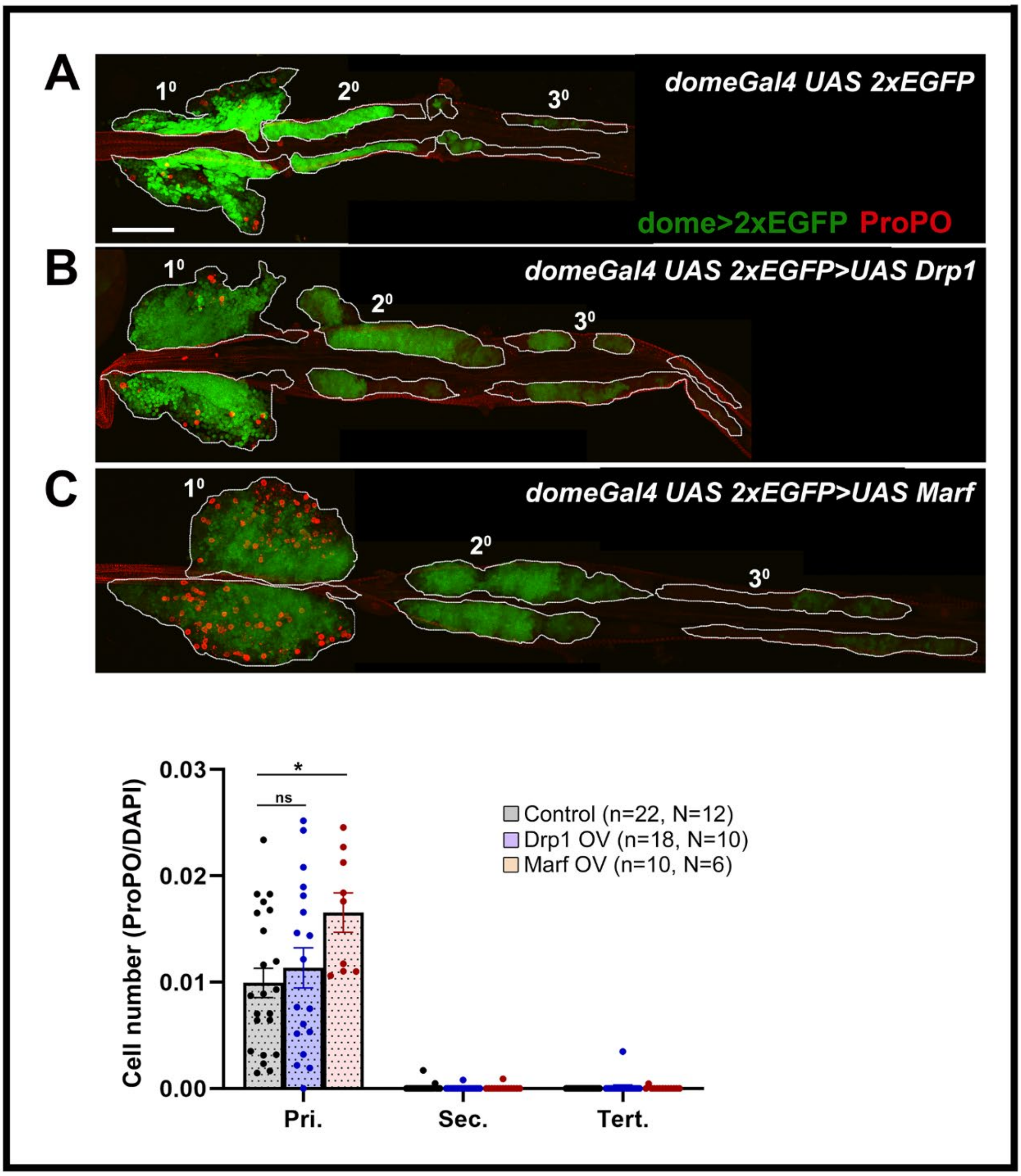

Supplementary Figure S6. Overexpression of Drp1 and Marf differentially affect crystal cell differentiation in *Drosophila* lymph gland

(A-C) ProPO staining marks crystal cells in control (*domeGal4 UAS 2xEGFP*) (A), Drp1 OV (*domeGal4 UAS 2xEGFP>UAS Drp1*) (B) and Marf OV (*domeGal4 UAS 2xEGFP>UAS Marf*) (C) whole mount of lymph gland primary, secondary and tertiary lobes. GFP marks the expression of prohemocyte marker Domeless. n represents number of individual lymph gland lobes analysed, and N represents number of larvae for each genotype.

Scale bar: 100 µm. Error bars represent SEM. Kruskal Wallis test was performed to determine statistical significance. *P<0.05, ns: statistically non-significant difference.

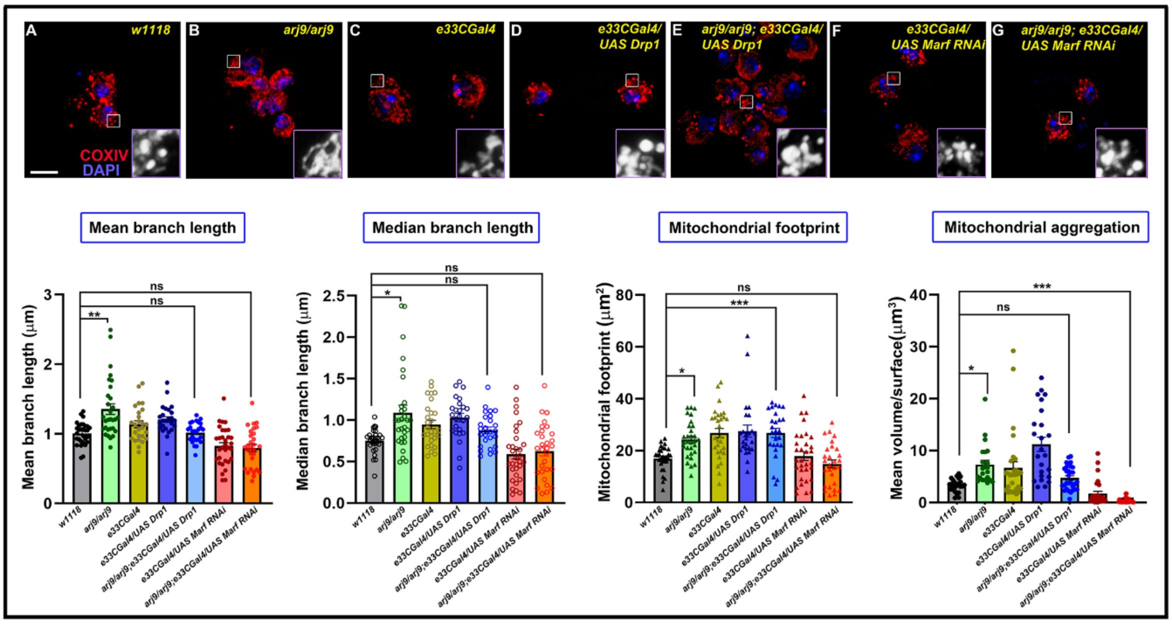

Supplementary Figure S7. Genetic interaction of *asrij* with Drp1 and *Marf* controls mitochondrial network architecture in circulatory hemocytes

(A-G) COXIV staining in control (w1118) (n=30 cells) (A), *asrij* KO (*arj9/arj9*) (n=30 cells) (B), *e33CGal4* (n=26 cells) (C), Drp1 OV (*e33CGal4/UAS Drp1*) (n=25 cells) (D), *asrij* KO Drp1

OV (*arj9*/*arj9*; *e33CGal4/UAS Drp1*) (n=26 cells) (E), *Marf* KD (*e33CGal4/UAS Marf RNAi*) (n=32 cells) (F), and *asrij* KO *Marf* KD (*arj9*/*arj9*; *e33CGal4/UAS Marf RNAi*) (n=30 cells) (G) circulatory hemocytes. Insets show magnified view of boxed region. Bar graphs show quantification of mitochondrial mean branch length, median branch length, mitochondrial footprint, and mitochondrial aggregation (mean volume/surface) in the same genotypes.

Scale bar: 10 μm. Error bars represent SEM. Kruskal Wallis test was used to determine statistical significance. *P<0.05, **P<0.01 and ***P<0.001. ns: statistically non-significant difference.

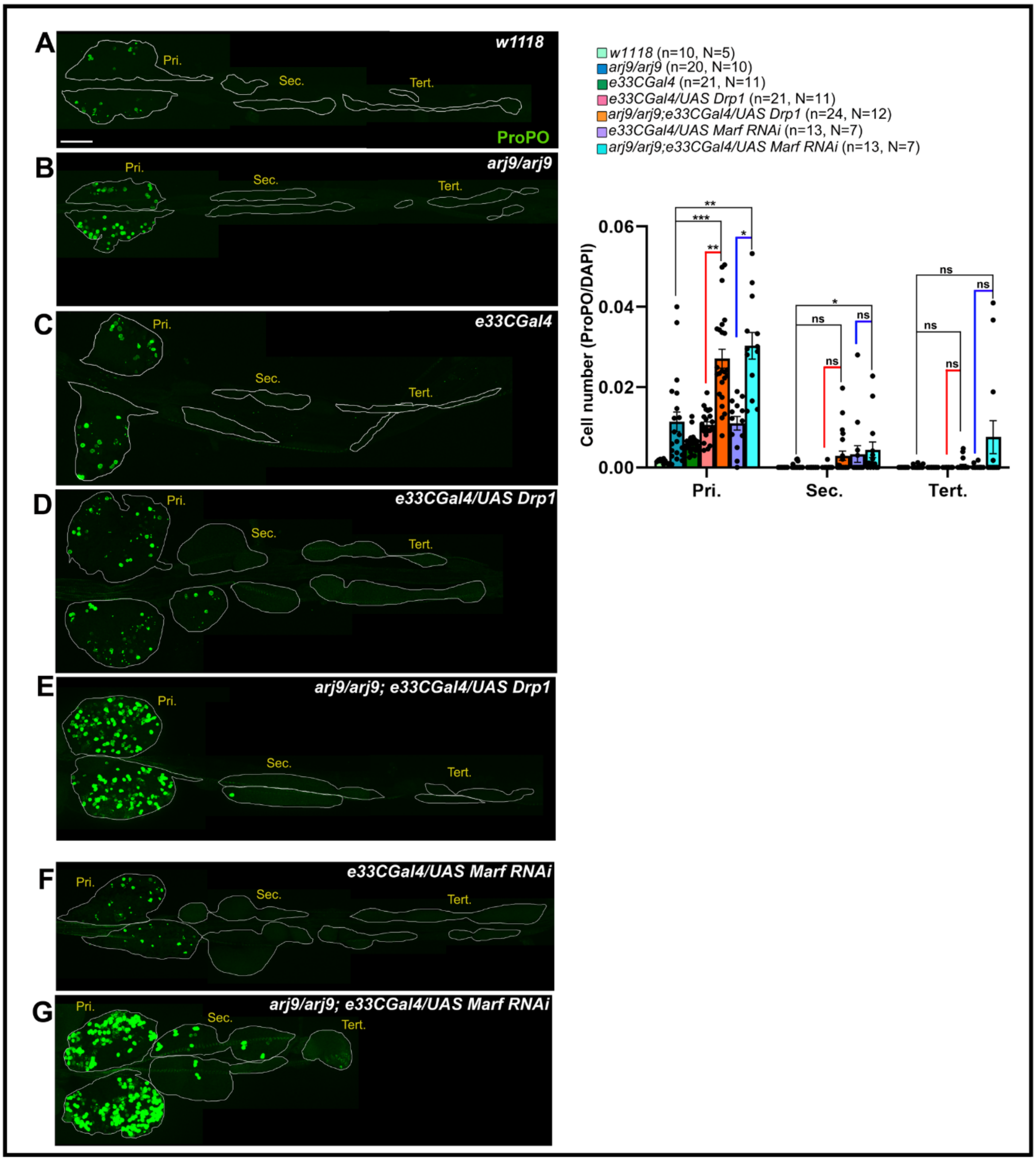

Supplementary Figure S8. Pan-hemocyte-specific genetic interaction of *asrij* with *Drp1* and *Marf* controls crystal cell differentiation in lymph gland

(A-E) Whole mount lymph gland showing ProPO expression to mark crystal cells in primary (Pri.), secondary (Sec.) and tertiary (Tert.) lobes of control (*w1118*) (A), *asrij* KO (*arj9/arj9*) (B), parental Gal4 control (*e33CGal4*) (C), Drp1 OV (*e33CGal4/UAS Drp1*) (D), *asrij* KO Drp1 OV

(*arj9*/*arj9*; *e33CGal4/UAS Drp1*) (E), *Marf* KD (*e33Cgal4/UAS Marf RNAi*) (F) and *asrij* KO *Marf* KD (*arj9*/*arj9*; *e33CGal4/UAS Marf RNAi*) (G) larvae.

(F) Bar graph shows quantification of mean crystal cell fraction in lymph gland lobes of indicated genotypes. Error bars indicate standard error of mean. n represents number of individual lymph gland lobes analysed, and N represents number of larvae for each genotype. Scale bar: 100 µm. Error bars represent SEM. Kruskal Wallis test was performed to determine statistical significance. *P<0.05, **P<0.01 and ***P<0.001; ns: statistically non-significant difference.

Supplementary Video S1. **Asrij regulates mitochondrial dynamics in hemocytes**

Time lapse video showing mitochondrial dynamics in control (*e33CGal4>UAS mito-GFP*) and asrij KD (*e33CGal4>UAS mito-GFP>UAS arj RNAi*) hemocytes.
